# Manipulating belief partially remedies the metamemory expectancy illusion in schema-based source monitoring

**DOI:** 10.3758/s13421-025-01757-2

**Published:** 2025-07-24

**Authors:** Marie Luisa Schaper, Ute J. Bayen

**Affiliations:** Institute for Experimental Psychology, Heinrich Heine University Düsseldorf, Düsseldorf, Germany

**Keywords:** Metamemory, Belief, Source monitoring, Schemas, Judgments of source

## Abstract

Metamemory illusions (i.e., false predictions of memory) are thought to arise from false a priori beliefs or from experiences made during study, such as processing fluency. The aim of the current research was to isolate the contribution of belief to metamemory by testing whether a correction of false beliefs can remedy a metamemory illusion. The authors focus on schema-based source monitoring, in which people show a metamemory *expectancy illusion* (e.g., Schaper et al., *Journal of Experimental Psychology: Learning, Memory, and Cognition, 45*(3), 470–496, 2019a). At study, people predict better source memory for items from expected sources (e.g., toothbrush in a bathroom), whereas actual source memory is better for items from unexpected sources (e.g., shampoo in a kitchen) or unaffected by expectations. In two source-monitoring experiments (*N* = 120/121), the authors tested whether the expectancy illusion could be remedied by correcting a priori belief. Participants studied items from expected and unexpected sources and made item-wise metamemory predictions about source memory. In both experiments, a manipulation to correct belief attenuated the expectancy illusion compared to a control group, but not to full remedy. Experiment 2 further revealed two distinct theoretical mechanisms underlying the partial persistence of the metamemory illusion: A partial inferential deficit, indicated by some participants failing to correct their belief, and a partial utilization deficit, indicated by participants failing to adequately use a corrected belief in metamemory judgments. The authors discuss competing influences of beliefs and experiences in metamemory judgment formation.

## Introduction

In metamemory research, it is widely accepted that people cannot directly access their actual memory representations when making metamemory judgments (e.g., Koriat, 1997; Rhodes, 2016). Instead, these judgments are considered inferential in nature. Theories of metamemory such as the cue-utilization approach (Koriat, 1997) typically adopt a dual-basis view, positing that item-wise metamemory judgments are influenced by in-the-moment experiences and/or by beliefs. For example, the experience of processing fluency during study (i.e., the subjective ease with which information is processed) affects metamemory (e.g., Besken, 2016; Undorf & Erdfelder, 2015; Undorf & Zimdahl, 2019). By contrast, beliefs involve inferences about memory based on characteristics of the material to be studied (e.g., Mueller & Dunlosky, 2017). For example, people believe that they will remember words printed in larger font better than words printed in smaller font even without prior study experience (Mueller et al., 2014). The relative contributions of experiences and beliefs in metamemory judgments are an ongoing area of investigation. In some situations, beliefs predominantly determine metamemory judgments (e.g., Hu et al., 2015; Mueller & Dunlosky, 2017; Mueller et al., 2013, 2014, 2016), whereas in others, experiences and beliefs jointly contribute (e.g., Frank & Kuhlmann, 2017; Jia et al., 2016; Schaper et al., 2019b, 2022).

Investigating *metamemory illusions* is a promising approach to discerning the effects of experiences and beliefs to particular metamemory judgments. Metamemory illusions arise when metamemory judgments are based on experiences and/or beliefs that are invalid predictors of future memory. In these cases, individuals misjudge the impact of certain factors on their memory (see review by Undorf, 2020). For example, the aforementioned belief that font size affects memory overestimates the actual effect of font size on memory (see meta-analyses by Chang & Brainerd, 2022; Luna et al., 2018). Based on the dual-basis view of metamemory, Koriat and Bjork (2006b) proposed that metamemory illusions may be remedied by altering experiences during study or by correcting erroneous beliefs (e.g., Blake & Castel, 2018; Wang et al., 2020; Yan et al., 2016). The effectiveness of these different remedial approaches may provide information on the relative contributions of experiences and beliefs to metamemory judgments.

The objective of the current study was to test whether correcting an illusory belief would either fully remedy the illusion, suggesting a dominant or exclusive contribution of belief, or partially remedy the illusion, suggesting a joint contribution of experience and belief, or fail to remedy the illusion, suggesting only a minor contribution of belief. In the following sections, we describe the metamemory illusion in question and outline the rationale for a belief-based remedy.

### The metamemory expectancy illusion in source monitoring

Source memory – remembering the source of information – is a central aspect of episodic memory (Johnson et al., 1993). Examples are remembering where you left your purse or who told you about a party. Typically, in everyday life, sources and the information they provide are related via schemas. Schemas are cognitive structures that organize knowledge and beliefs about the world (cf. Alba & Hasher, 1983). For example, based on schemas, it is expected for a US Republican politician to advocate for low corporate taxes, whereas it would be unexpected for a Republican to oppose the right to bear arms. Critically for the present purpose, source memory and metamemory are oppositely affected by schema-based expectations that sources evoke about the information they provide.

In the laboratory, the effects of schema-based expectations on source memory and metamemory are typically studied as follows (see review by Kuhlmann et al., 2021). Participants study items of information that originate from either a schematically expected source or from a schematically unexpected source. After study, participants complete a source-monitoring test (cf. Johnson et al., 1993). In this test, they are presented with studied items and distractors and decide, for each item, whether it was presented at study and, if so, which source it originated from. Previous research employed various source–item materials, including persons behaving in a stereotype-consistent, expected or in a stereotype-inconsistent, unexpected manner (e.g., concerning profession: Arnold et al., 2013; Bayen & Kuhlmann, 2011; Bayen et al., 2000; Besken & Gülgöz, 2008; Dodson et al., 2008; Hicks & Cockman, 2003; Konopka & Benjamin, 2009; Kuhlmann et al., 2012; Shi et al., 2012; Spaniol & Bayen, 2002; Wulff & Kuhlmann, 2020; social behavior and appearance: Bell et al., 2012; Ehrenberg & Klauer, 2005; Kranz et al., 2019; Kroneisen & Bell, 2013; Kroneisen et al., 2015; Mieth et al., 2021; Sherman & Bessenoff, 1999; Sherman et al., 1998; gender: Kleider et al., 2008; Marsh et al., 2006; age: Kuhlmann et al., 2016; or partisanship: Mather et al., 1999), and scenes with expected or unexpected objects (e.g., “toothbrush in the bathroom” vs. “shampoo in the kitchen”; e.g., Bayen et al., 2000; Küppers & Bayen, 2014; Lew & Howe, 2017; Schaper & Bayen, 2021; Schaper et al., 2019a, 2019b, 2022, 2023a, 2023b). We used the latter manipulation in the current study.

Schema-based expectations affect source memory and metamemory differentially. Source memory has been found to be either unaffected by schema-based expectations or better for unexpected sources (i.e., *inconsistency effect*; see review by Kuhlmann et al., 2021).^1^ However, when asked to predict their schema-based source memory, people are under the illusion that source memory is better for expected than unexpected source–item pairs (i.e., they incorrectly predict an *expectancy effect*; Mieth et al., 2021; Schaper & Bayen, 2021; Schaper et al., 2019a, 2019b, 2022, 2023a, 2023b).

Item-wise predictions of source memory are referred to as *Judgments of Source* (JOSs, cf. Carroll et al., 1999; analogous to *Judgments of Learning*, JOLs, which are predictions of item memory, see review by Rhodes, 2016). In JOSs, participants predict the likelihood of remembering the source of an item at later test (see review by Kuhlmann & Bayen, 2016). The *metamemory expectancy illusion* is the dissociative pattern of results in source memory (null effect or inconsistency effect) and JOSs (expectancy effect). This illusion is robust and emerged with different source–item materials (with expected and unexpected scene–object pairs, Schaper & Bayen, 2021; Schaper et al., 2019a, 2019b, 2022, 2023a, 2023b; and with trustworthy- and untrustworthy-looking cheaters and cooperators, Mieth et al., 2021). The illusion is also relevant because it affects study behavior: Participants chose unexpected source–item pairs more often for re-study because they incorrectly predicted worse source memory for them (Schaper & Bayen, 2021; Schaper et al., 2023a).

### The role of belief in the metamemory expectancy illusion

In the experiments reported here, we aimed to test the contribution of belief to the expectancy illusion using a belief-based remedy. While prior evidence suggests that belief contributes to the illusion, the extent of the contribution remains unclear. Schaper et al. (2019b) showed that participants held an expectancy belief even before study, which contributed to the illusion. Specifically, participants with stronger a priori belief in an expectancy effect were more likely to express an expectancy effect in their item-wise JOSs. However, this study could not rule out an additional contribution of experience to the expectancy illusion. Expected source–item pairs were processed more fluently than unexpected pairs (see also Sherman et al., 1998), which participants seemed to mistakenly associate with enhanced source memory. Previous remedies of the expectancy illusion have exclusively involved a manipulation that affected both experiences and belief, namely delaying JOSs (Schaper et al., 2022, 2023a). Delayed judgments improve the accuracy of item-wise metamemory predictions (see meta-analysis by Rhodes & Tauber, 2011) and can remedy metamemory illusions (Luna et al., 2018; Metcalfe & Finn, 2008; Yang et al., 2017). The beneficial effect of delaying judgments has traditionally been attributed to experiences, namely a shift from reliance on processing fluency to the more predictive retrieval fluency (i.e., the subjective ease of retrieving information; Benjamin & Bjork, 1996; Koriat & Bjork, 2006a, 2006b; Koriat & Ma’ayan, 2005). In our prior source-monitoring studies (Schaper et al., 2022, 2023a), JOSs were not made immediately at first study, but were delayed until a second study phase in which only the items, not the sources, were presented. This delay was effective only when participants accessed retrieval fluency during judgment formation; that is, they retrieved the sources before rendering the judgments. However, the delay also positively influenced belief about schema-based source monitoring, presumably because participants noticed during source retrieval that their source memory was better for unexpected source–item pairs. Schaper et al., (2022, 2023a) concluded that delaying JOSs effectively combined experience-based and belief-based mechanisms to remedy the expectancy illusion.

One might object that delayed JOSs after source retrieval reflect metamemory at the time of test rather than at the time of study (cf. Koriat & Bjork, 2006a; Schaper et al., 2022). To fully remedy the expectancy illusion, an intervention should enable accurate predictions of source memory already during the first encounter with source–item information, that is, at the time of study. We, therefore, sought to remedy the expectancy illusion by changing participants’ a priori belief before study with a purely belief-based intervention. Despite the aforementioned evidence for a role of belief in the expectancy illusion, no prior study has examined the influence of belief on JOSs independently of experience.

If a purely belief-based intervention remedies the illusion, this would indicate that people can apply a changed belief about memory in their JOSs. If belief plays the dominant role in the expectancy illusion, correcting belief alone should remedy the illusion at study. However, it may be the case that belief does not play the dominant role in the expectancy illusion. Two theoretical explanations have been proposed for why people may not apply corrected beliefs in metamemory judgments. These are an *inferential deficit* (Hertzog et al., 2009; Mueller et al., 2015) and a *utilization deficit* (Mueller et al., 2015). Inferential deficit means that people hold inaccurate metamemory beliefs due to invalid inferences even after an intervention to correct belief. In the schema-based source-monitoring paradigm, people may maintain the erroneous belief that expected sources are more easily remembered. A utilization deficit, by contrast, means that people hold accurate beliefs, but use them deficiently. People may not apply a corrected belief in an inconsistency effect to their JOSs because other factors, such as the experience of processing fluency, also play a role in these judgments.

### The current experiments

The aim of the current experiments was to test the contribution of belief to the expectancy illusion in JOSs in source monitoring using an exclusively belief-based remedy. To achieve this, we aimed to correct people’s erroneous belief about an expectancy effect on source memory. We conducted two source-monitoring experiments with expected and unexpected source–item pairs. To remedy the illusion, we experimentally manipulated participants’ belief toward an inconsistency effect before study.

In the source-monitoring paradigm, participants have been shown to believe in an illusory expectancy effect on source memory prior to study (cf. Schaper et al., 2019b). In both experiments, we implemented two groups to test whether a belief manipulation affected JOSs during study. The *control groups* received no information before the experiments and thus, were expected to show a naïve expectancy belief (cf. Schaper et al., 2019b) and the established expectancy illusion on item-wise JOSs (cf. Mieth et al., 2021; Schaper & Bayen, 2021; Schaper et al., 2019a, 2019b, 2022, 2023a, 2023b). By contrast, the *experimental groups* were provided with detailed information about the paradigm and the expectancy illusion, including information about the inconsistency effect on source memory, the illusory expectancy effect on metamemory, and the role of experiences in this effect. Similar belief manipulations have been shown to be effective at eliminating other illusions on item-wise metamemory judgments, such as the font-size illusion (Blake & Castel, 2018; Wang et al., 2020).

Critically, in a study by Yan et al. (2016), the effectiveness of belief manipulations depended on the strength of the manipulation. When more information was provided, participants more likely accepted the manipulation. In a previous experiment (Schaper et al., 2019a, Experiment 4), a simple and brief belief manipulation merely eliminated the expectancy belief toward a null belief, but did not achieve an inconsistency belief. This previous experiment had the additional limitation that we only asked participants for a global belief assessment and did not assess item-wise JOSs, making it impossible to disentangle the contributions of belief in contrast to experience to the expectancy illusion. In the experimental group of the current experiments, we implemented a stronger manipulation of belief than in Schaper et al. (2019a).^2^ Participants received a detailed text and accompanying figures with explanations of the research investigating the expectancy illusion (available in the Open Science Framework). In both experiments, we assessed item-wise JOSs at study. In Experiment 2, we additionally assessed belief about the effect of expectations on source memory before study so that we were able to determine directly if a correction of belief took place as a result of the manipulation.

If belief is the driving factor behind the expectancy illusion, then a corrected belief should lead to a full remedy of the illusion. That is, we would expect the experimental group to show an inconsistency belief before study and an inconsistency effect on item-wise JOSs in alignment with the inconsistency effect on actual source memory. However, it is also possible that the illusion on JOSs may only be partially remedied or not at all. A partial remedy would manifest as an attenuated expectancy effect or a null effect on JOSs in the experimental group. The absence of a remedy would manifest as an equally strong expectancy effect on JOSs in both groups.

A partial or full persistence of the expectancy illusion may be due to participants showing an inferential deficit and/or a utilization deficit of belief. Specifically, an inferential deficit would manifest as belief before study not being fully corrected towards an inconsistency belief in the experimental group (i.e., showing a null belief or an expectancy belief). A utilization deficit would mean that participants fail to apply or only partially apply their (corrected) inconsistency belief during JOSs. In Experiment 2, we tested whether belief influenced the strength of the effect of expectancy on JOSs. If participants showed a full utilization deficit, a corrected belief should not influence the strength of the effect of expectancy on JOSs, resulting in no remedy of the expectancy illusion. This would suggest that belief plays a minimal role and the expectancy illusion is primarily driven by experiences. If participants showed a partial utilization deficit, the corrected belief should influence the strength of the effect of expectancy, but the expectancy illusion should partially persist (i.e., JOSs should show a null effect or attenuated expectancy effect). This would suggest a joint contribution of belief and experience to the illusion. Finally, it is likely that experiencing one’s own source memory during the source-monitoring test corrects one’s belief (cf. Schaper et al., 2019a). In this case, the control group should show a difference between the belief they hold before study and the belief they hold after test. For the experimental group, by contrast, experiences during test should not change belief if belief is already fully corrected before study. In this group, the correct inconsistency belief should, therefore, manifest to the same extent in belief before study and after test. In addition to JOSs at study (both experiments) and belief before study (Experiment 2), we therefore assessed belief after the source-monitoring test.

## Experiment 1

### Method

#### Data availability, transparency, and openness

We provide supplementary analyses, the materials, data, and analysis code for both experiments in the Open Science Framework (https://osf.io/ze3qb/). The repository is cited in the reference section. The experiments and analysis plan were not preregistered.

#### Participants

Both experiments were approved by the local ethics committee. We wanted to be able to detect an inconsistency effect on JOSs in the experimental group. To detect a remedy in the form of a small inconsistency effect of size *d*_z_ = 0.33 (one-sided, within-subjects; cf. Schaper et al., 2019a) with α = 0.05 and a power of 0.80, 59 participants are needed. We included 60 participants in each group to completely counterbalance the materials. We recruited 120 students who were native speakers of German (80 female, 40 male) on the campus of Heinrich-Heine-Universität Düsseldorf, Germany. Students of psychology were first-year students. Data collection took place in our laboratory between 1 April 2019, and 27 May 2019. We excluded and replaced one participant because they terminated participation early. Age ranged between 18 and 34 years (*M* = 21.52, *SE* = 0.27). Participants were compensated with 8 € or partial course credit.

#### Design and material

The experiment had a 2 × 2 mixed-factorial design with the between-subjects factor belief manipulation (experimental, control), the within-subjects factor source–item expectancy (expected, unexpected), and source memory and metamemory as dependent variables. Participants were alternatingly assigned to the two groups.

##### Items and sources

The item materials and counterbalancing scheme were the same as in previous experiments (Schaper & Bayen, 2021; Schaper et al., 2019a, 2019b, 2022, 2023a). That is, the total item pool consisted of 96 object labels, 48 of which were expected for a kitchen and unexpected for a bathroom (hitherto referred to as *kitchen items*, e.g., “frying pan”) and the other 48 expected for a bathroom and unexpected for a kitchen (*bathroom items*, e.g., “toothbrush”). In a norming study (reported in detail by Schaper et al., 2019a), items were rated regarding their expectancy for occurring in a bathroom or a kitchen on a 5-point Likert scale from 1 (*very unexpected*) to 5 (*very expected*). The selected items (between one to six syllables) had a mean rating of 4.49 (*SE* = 0.03) for their expected source and a mean rating of 1.10 (*SE* = 0.01) for their unexpected source. The item pool was split into three item lists, each containing 16 kitchen items and 16 bathroom items (i.e., 32 items per list) matched in mean expectancy for the expected source, mean inconsistency for the unexpected source, number of syllables, and word frequency (according to German word norms by the University of Leipzig). Detailed statistics for the three item lists are in the Open Science Framework. At study, items from one list were presented with the bathroom source, and items from a second list were presented with the kitchen source. At test, items from the third list served as distractors. This assignment of the lists was counterbalanced within groups.

##### Belief manipulation

For the experimental group, we created a text that explained the expectancy illusion in source monitoring in detail (i.e., the expectancy effect on metamemory and the inconsistency effect on source memory). Participants were informed that they would receive information about the research they were participating in and that they should read the instructions carefully and would later be tested on them. An expectancy belief may, in part, be based on the belief that processing fluency benefits memory (cf. Mueller & Dunlosky, 2017). To counteract this possibility, we discounted processing fluency in the manipulation: We informed participants that although they might find it subjectively easier to learn the expected trials, source memory is generally better for unexpected trials. We ensured that the text was understandable for non-psychologists via a pilot study in which ten participants answered questions on text comprehension. A professional actor read the text for an audio recording that was 7 min and 13 s in length. While listening to the recording via headphones, participants saw the same text and accompanying figures to illustrate the key concepts (to which the text referred where appropriate) on the computer screen. The manipulation materials in the original German version and an English translation are provided in the Open Science Framework.

#### Procedure

##### Belief manipulation

Up to four participants per session were seated in individual computer booths and signed informed consent. In the experimental group, belief was manipulated at the beginning of the experiment. That is, the text and figures were presented on-screen and the corresponding audio recording was presented simultaneously via headphones. To approximately equate the interval between the start of the experiment and the start of the study phase between groups, the control group started the experiment with a filler task (arithmetic equations) that lasted 7 min and 13 s.

As a manipulation check, we tested participants’ understanding of the information in the experimental group. They were asked (1) whether memory was better for items presented with the expected or unexpected source, and (2) whether it felt subjectively easier to study items presented with the expected or unexpected source. Within each question, the order of expected/unexpected was counterbalanced. The original German questions and their translation are provided in the Open Science Framework. Participants could proceed with the experiment only if they answered both questions correctly. Otherwise, the manipulation was repeated and checked once more. One participant first responded incorrectly to the second question and responded correctly to both questions after repetition of the manipulation.^3^ The control group did not receive any questions.

##### Study phase

All participants were then instructed to study the source–item pairs and to memorize both the items and their sources. They were further instructed to render a JOS after studying each pair (assuming perfect item memory). At study, items (in standard German capitalization) and their sources (in all capitals, i.e., “in the BATHROOM” or “in the KITCHEN”) were presented in white letters on black background, one pair at a time for 4 s. Participants studied 32 expected and 32 unexpected source–item pairs in an order that was randomized by participants. After each pair, on a separate screen, they rendered a self-paced JOS to the prompt “Likelihood that you will later remember in which ROOM (KITCHEN or BATHROOM) this object was located?” (room order counterbalanced). They made the JOS on a scale from 0% (*definitely will not remember*) to 100% (*definitely will remember*) using the computer keyboard.

##### Test phase

Immediately after study, participants received instructions for the source-monitoring test. Their task was to indicate whether an item had been presented with the bathroom source, the kitchen source, or had not been presented at study. The 64 studied items and 32 distractors were presented one at a time in random order. Below each item, two gray response boxes, labeled “in the BATHROOM” and “in the KITCHEN” in black text, were presented side by side (location counterbalanced). A third response box was presented centered beneath the other two and labeled “was not presented.” To respond, participants clicked on the corresponding response box with the computer mouse. Responses were self-paced.

##### Belief judgments

After the test phase, participants rendered two self-paced belief judgments. They were asked to postdict their own source memory separately for expected and unexpected items. For each item type, they indicated how many sources they thought they had remembered on a scale from 0% (*did not remember any of the rooms*) to 100% (*remembered all of the rooms*). Lastly, participants were debriefed and compensated. The total duration of the experiment was about 40 min.

### Results and discussion

The data and supplementary analyses for both experiments can be found in the Open Science Framework at https://osf.io/ze3qb/. Alpha was set to 0.05 for all analyses. We first report results on source memory against which metamemory can be compared. Then, we report the critical results on metamemory, that is, JOSs and belief judgments after test.

#### Source memory

##### Measuring source memory

**Fig. 1:**
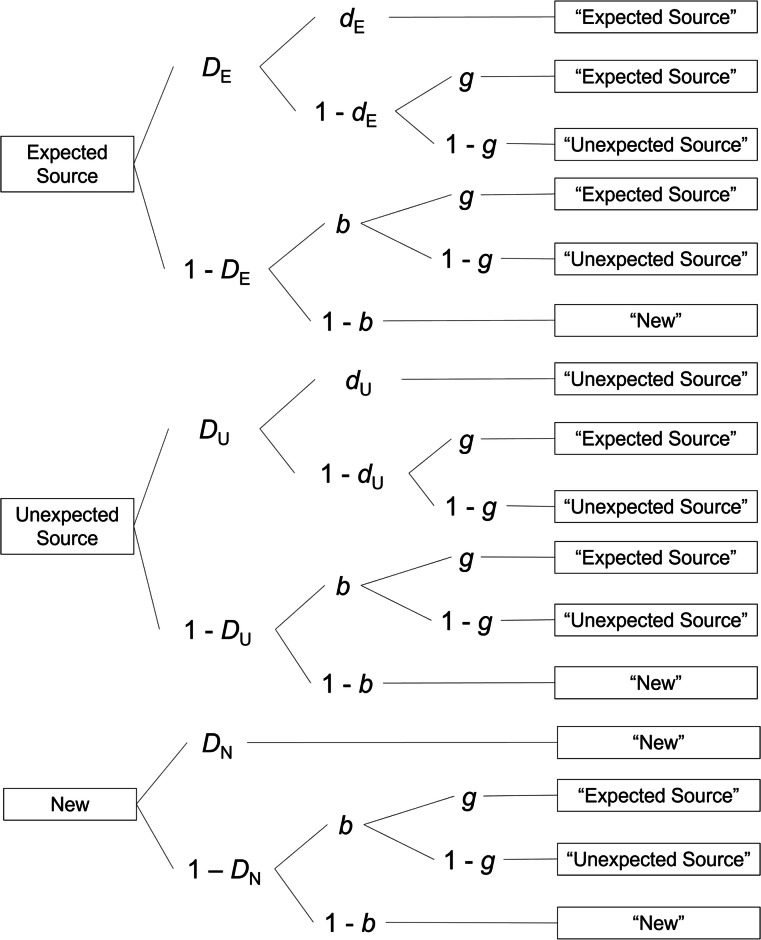
Multinomial processing-tree model of source monitoring. *D*_E_ = probability of recognizing an item that was presented with the expected source; *D*_U_ = probability of recognizing an item that was presented with the unexpected source; *D*_N_ = probability of knowing that a new item is new; *d*_E_ = probability of remembering that an item was presented with the expected source; *d*_U_ = probability of remembering that an item was presented with the unexpected source; *g* = probability of guessing that an item was presented with the expected source; *b* = probability of guessing that an item was old. This version of the model assumes source guessing is equal for recognized and non-recognized items. Adapted from “Source Discrimination, Item Detection, and Multinomial Models of Source Monitoring,” by U. J. Bayen, K. Murnane, and E. Erdfelder, 1996, *Journal of Experimental Psychology: Learning, Memory, and Cognition, 22,* p. 202, Fig. 3. Copyright 1996 by the American Psychological Association

Performance in source-monitoring tasks is determined by multiple cognitive processes including source memory and source guessing (e.g., Batchelder & Riefer, 1990; Murnane & Bayen, 1998). It is important to measure source memory independent of source guessing because these processes are differentially affected by schema-based expectations (Bayen et al., 2000; see review by Kuhlmann et al., 2021). Whereas behavioral measures of source-monitoring performance confound source memory and source guessing (Bayen et al., 1996; Murnane & Bayen, 1996, 1998), multinomial processing tree (MPT) modeling allows for separate measurement of processes that underlie performance in source-monitoring tasks. MPT models assume discrete memory states (i.e., information is remembered when it passes a threshold and is not remembered when it does not pass the threshold), which is supported by empirical studies (e.g., Klauer & Kellen, 2010; Schütz & Bröder, 2011). Figure 1 shows the two-high threshold MPT model of source monitoring (Bayen et al., 1996), which has been experimentally validated to provide measures of item memory (i.e., discriminating studied items and distractor items, parameter *D*), source memory (i.e., remembering the source of a recognized item, parameter *d*), old/new guessing (i.e., guessing old or new in the absence of item memory, parameter *b*), and source guessing (i.e., guessing the source in the absence of source memory, parameter *g*; for experimental validation, see Bayen & Kuhlmann, 2011; Bayen et al., 1996). Parameters are estimated from the observed frequencies of different response types in the source-monitoring test (see Table 1 for the frequencies).

**Table 1:** Response frequencies in the source-monitoring test

Experiment	Group	Trial type		Response	
			“Expected”	“Unexpected”	“New”
1	Control	Expected source	1,138	323	459
	(*n* = 60)	Unexpected source	405	987	528
		New	137	70	1,713
	Experimental	Expected source	1,107	311	502
	(*n* = 60)	Unexpected source	390	1,045	485
		New	166	68	1,686
2	Control	Expected source	1,135	293	492
	(*n* = 60)	Unexpected source	435	1,016	469
		New	165	76	1,679
	Experimental	Expected source	1,202	316	434
	(*n* = 61)	Unexpected source	477	1,011	464
		New	238	93	1,621

Control group: participants did not receive a belief manipulation; experimental group: participants received the belief manipulation before study

The first processing tree in Fig. 1 pertains to items presented with the expected source, the second tree to items presented with the unexpected source, and the third tree to distractor items. Participants recognize an item that was presented with the expected source as old with probability *D*_E_. Given that they recognize the item, they remember that the item was presented with the expected source with probability *d*_E_ (i.e., source memory). Conversely, with probability 1 – *d*_E_, participants do not remember the source of the item and must guess a source. In that case, participants guess that an item was presented with the expected source with probability *g*, and guess that the item was presented with the unexpected source with the complementary probability 1 – *g*. Participants do not recognize the item with probability 1 – *D*_E_, in which case they must guess the old/new status of the item. They guess that the item was old with probability *b*, and guess that it was new with the complementary probability 1 – *b*. If they guess the item to be old, they either guess that the item was presented with the expected source with probability *g*, or guess that it was presented with the unexpected source with probability 1 – *g*. Analogously, in the second tree, the probability of recognizing an item presented with the unexpected source is *D*_U_ and the probability of remembering its source is *d*_U_. The third tree, *D*_N_ is the probability of knowing that a distractor item is new. We used the customary equality restrictions that *D*_E_ = *D*_U_ = *D*_N_ to obtain a mathematically identifiable model (Bayen et al., 1996).

For parameter estimation, we used the R package *TreeBUGS* (Heck et al., 2018; R Core Team, 2020) and the Bayesian-hierarchical latent trait approach (Klauer, 2010). From an overarching multivariate normal distribution of probit-transformed parameters, participant parameters are drawn. Given the data, the a priori distribution is updated to a posterior distribution for the parameters using Bayes’ theorem. From the posterior distribution, samples are drawn by the Markov chain Monte Carlo algorithm. Thereby, we obtained parameter estimates and corresponding 95% Bayesian credibility intervals (BCIs) in which the true parameter can be found with 95% confidence.

We calculated a model for both groups using the uninformative default prior implemented in *TreeBUGS*. Group membership was included as a binary predictor for all parameters (cf. Heck et al., 2018). We obtained three chains with 500,000 samples (250,000 burn-in samples) each and retained every 50th sample. The Gelman-Rubin statistic $\widehat{R}$ (Gelman & Rubin, 1992) indicates good parameter convergence if $\widehat{R}$ < 1.01, which was the case for all parameters. Model fit statistics *T*_1_ and *T*_2_ (Klauer, 2010) indicate good model fit via non-significant test results. *T*_1_ measures the distance between observed and expected mean frequencies; *T*_2_ measures the distance between observed and expected covariances (see Heck et al., 2018). The model fit the data well (*T*_1_: *p* = 0.384, *T*_2_: *p* = 0.402). Group did not affect any of the parameters as all 95% BCIs for the group effects included zero (Effect_*D*_ = 0.02 [−0.06, 0.10], Effect_*dE*_ = 0.06 [−0.34, 0.52], Effect_*dU*_ = 0.08 [−0.10, 0.26], Effect_*g*_ < 0.01 [−0.15, 0.15], Effect_*b*_ = 0.04 [−0.08, 0.17]). In particular, these results indicate that item memory was affected by neither group nor expectancy. Table 2 shows the full set of parameter estimates.

**Table 2:** Parameter estimates from the multinomial processing-tree modeling

	Experiment 1		Experiment 2	
	Control	Experimental	Control	Experimental
*D*_E_ = *D*_U_ = *D*_N_	.64 [.60,.68]	.63 [.59,.67]	.61 [.57,.65]	.63 [.58,.67]
*d*_E_	.27 [.05,.51]	.30 [.07,.57]	.25 [.07,.49]	.37 [.13,.61]
*d*_U_	.63 [.52,.73]	.69 [.59,.78]	.67 [.58,.74]	.63 [.54,.71]
*b*	.26 [.21,.32]	.29 [.23,.35]	.34 [.27,.42]	.29 [.22,.36]
*g*	.65 [.56,.74]	.65 [.56,.74]	.66 [.59,.74]	.63 [.55,.71]

Parameter estimates are probabilities in the interval [0, 1]. Control group: participants did not receive a belief manipulation; experimental group: participants received the belief manipulation before study. *D*_E_/*D*_U_ = probability that an item from the expected/unexpected source was recognized as old. *D*_N_ = probability that a participant knew that a new item was new. *d*_E_/*d*_U_ = probability that a participant remembered the expected/unexpected source of an item. *b* = probability that a participant guessed that an item was old. *g* = probability that a participant guessed that an item had been presented with the source for which it was expected. 95% Bayesian credibility intervals are in brackets

##### Source-memory results

**Fig. 2:**
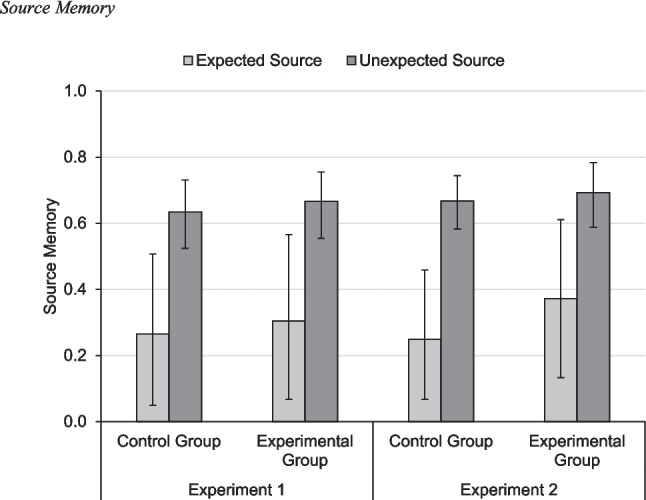
Source memory. Probability estimates of the source-memory parameter *d* as obtained by multinomial-processing-tree modeling. Control group: participants did not receive a belief manipulation; experimental group: participants received a belief manipulation before study. Error bars denote 95% Bayesian credibility intervals

We expected inconsistency effects on source memory (for a review, see Kuhlmann et al., 2021). Therefore, we compared the parameters *d*_E_ and *d*_U_ (i.e., the parameters measuring source memory for expected and unexpected items, respectively). As shown in Fig. 2, both groups showed numerical inconsistency effects. To test the overall inconsistency effect, we sampled the difference between the source-memory parameters, Δ*d* = *d*_U_ – *d*_E_. A positive difference indicates better source memory for items from the unexpected source and thus an inconsistency effect. A difference was considered reliable when the 95% BCI of the difference did not contain zero. As expected, the inconsistency effect was reliable, Δ*d* = 0.38 [0.14, 0.63].^4^ In the next paragraphs, we compare source memory with JOSs during study and with belief judgments after test.

#### Metamemory judgments

**Fig. 3:**
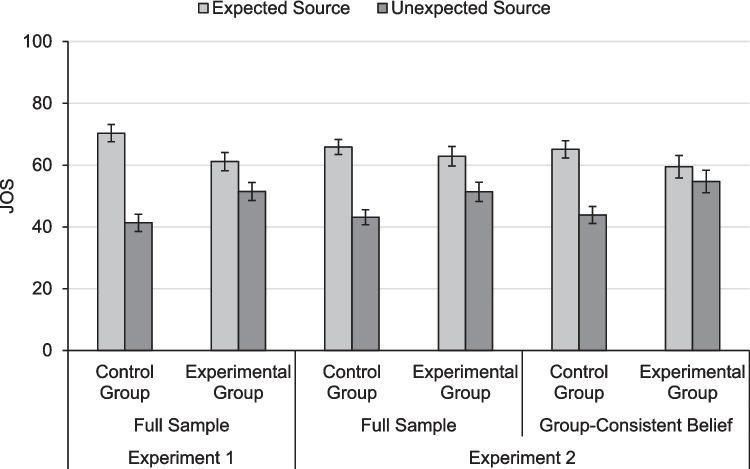
Mean judgments of source (JOS, 0–100%). For both experiments, we report mean JOSs for the full sample. In Experiment 2, we also report mean JOSs for the subset of participants who showed a group-consistent belief before study (i.e., expectancy belief in the control group, inconsistency belief in the experimental group). Control group: participants did not receive a belief manipulation; experimental group: participants received a belief manipulation before study. Error bars denote 95% within-subjects confidence intervals (Loftus & Masson, 1994), which are adjusted for the respective within-subjects comparison between expected and unexpected trials

**JOSs.** Figure 3 shows the means and 95% confidence intervals (CIs) for JOSs. We calculated a 2 × 2 mixed ANOVA with the factors expectancy and belief manipulation on JOSs. There was no main effect of belief manipulation, *F*(1, 118) = 0.04, *p* = 0.840, η_p_^2^ < 0.01. There was a main effect of expectancy on JOSs, *F*(1, 118) = 91.60, *p* < 0.001, η_p_^2^ = 0.44. That is, participants predicted better source memory for expected than unexpected source–item pairs. Critically, there was a hybrid two-way interaction, *F*(1, 118) = 22.97, *p* < 0.001, η_p_^2^ = 0.16. This interaction indicated that the expectancy effect was stronger in the control group than in the experimental group, even though it was significant in both the control group, *t*(59) = 10.39, *p* < 0.001, *d*_z_ = 1.34, and the experimental group, *t*(59) = 3.31, *p* = 0.002, *d*_z_ = 0.43. Thus, both groups showed illusory expectancy effects on JOSs that were doubly dissociated from the inconsistency effect on source memory. Hence, the belief manipulation attenuated the expectancy illusion, but not to full remedy.

This partial remedy of the expectancy illusion resulted from fewer participants falling prone to the illusion in the experimental group than the control group. In the control group, the vast majority of participants, namely 55 out of 60, predicted an expectancy effect (i.e., higher mean JOSs for expected than unexpected source–item pairs). Only four predicted an inconsistency effect (i.e., higher mean JOSs for unexpected than expected pairs), and one a null effect (i.e., exact equal mean JOSs for expected and unexpected pairs). By contrast, in the experimental group, 40 out of 60 participants predicted an expectancy effect and 20 predicted an inconsistency effect. The proportion of participants who predicted an expectancy effect was smaller in the experimental group than in the control group, *z* = 3.37, *p* < 0.001.^5^

These results suggest that belief was altered by the belief manipulation prior to study to some degree and did influence JOSs, but that experiences of processing fluency additionally contributed to the expectancy illusion on JOSs (cf. Schaper et al., 2019b). The results are compatible with both a partial inferential deficit and a partial utilization deficit regarding belief. In terms of an inferential deficit, it is possible that some participants did not adopt the correct inconsistency belief, for example, because they were resistant to changing their pre-existing expectancy belief (cf. Schaper et al., 2019b). Further, it is possible that despite acquiring the accurate belief, participants may have used it deficiently. Schaper et al. (2019b) showed that the naïve expectancy belief (which, in the current experiment, was supposedly held by the control group) predicted the expectancy effect on JOSs. It is possible that participants in the experimental group did adopt the correct inconsistency belief, but then did not use it as strongly to inform their JOSs. We address these possibilities in Experiment 2.

##### Belief after test

**Fig. 4:**
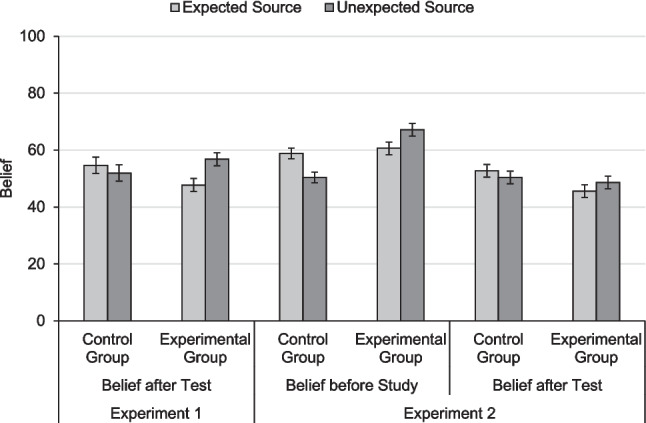
Mean belief judgments (0–100%). For both experiments, we report belief judgments elicited after test. For Experiment 2, we also report belief judgments elicited before study. Control group: participants did not receive a belief manipulation; experimental group: participants received a belief manipulation before study. Error bars denote 95% within-subjects confidence intervals (Loftus & Masson, 1994), which are adjusted for the respective within-subjects comparison between expected and unexpected trials

Figure 4 shows the means and 95% CIs for belief judgments. We calculated a 2 × 2 mixed ANOVA with the factors expectancy and belief manipulation on belief judgments after test. There was no main effect of belief manipulation, *F*(1, 118) = 0.12, *p* = 0.733, η_p_^2^ < 0.01, and no main effect of expectancy, *F*(1, 118) = 3.06. *p* = 0.083, η_p_^2^ = 0.03, but a two-way interaction, *F*(1, 118) = 10.42, *p* = 0.002, η_p_^2^ = 0.08. The control group showed no effect of expectancy on belief, *t*(59) = 0.94, *p* = 0.349, *d*_z_ = 0.12. The participants of the control group did not correctly postdict their own source memory, because they did not show an inconsistency belief. By contrast, the experimental group did show an inconsistency effect on belief, *t*(59) = 4.00, *p* < 0.001, *d*_z_ = 0.43, which mirrored the inconsistency effect on source memory in this group. These results suggest that both the belief manipulation as well as test experience positively affected belief. However, it is necessary to compare belief after test to belief before study, which we did in Experiment 2.

## Experiment 2

Experiment 2 had two aims. First, we sought to replicate the results of Experiment 1. Second, we aimed to further test the possible reasons for the partial persistence of the expectancy illusion on JOSs, specifically, whether it reflects an inferential deficit or a utilization deficit. To this end, we closely replicated the procedure of Experiment 1 but added belief judgments prior to the study phase in both groups. This addition enabled us to disentangle whether participants showed a partial inferential deficit (i.e., failed to correct their belief) or a partial utilization deficit (i.e., failed to apply their corrected belief during judgment).

### Method

#### Participants

The power considerations and inclusion criteria were the same as in Experiment 1. We recruited 121 new participants (70 female, 51 male) on the campus of Heinrich-Heine-Universität Düsseldorf, Germany. Data collection took place in our laboratory between 31 March 2025 and 16 April 2025. We alternatingly assigned 60 participants to the control group and 61 participants to the experimental group. We excluded and replaced one participant because of technical failure. Age ranged between 17 and 34 years (*M* = 21.04, *SE* = 0.24).^6^ Participants were compensated with 10 € or partial course credit.

#### Design, material, and procedure

The design, materials, and procedure were identical to those in Experiment 1, with the following exception. Before the instructions for the study phase (i.e., after the filler task in the control group and after the manipulation checks in the experimental group), participants rendered two self-paced belief judgments. At this point, they were informed that they would be shown object labels along with one of two rooms in which the object was located (kitchen or bathroom, with order counterbalanced) and that 50% of the objects would appear with the expected room, whereas the other 50% would appear with the unexpected room. Then, they were asked to predict their own source memory for expected and unexpected items separately. For each item type, they indicated how many sources they believed they would remember on a scale from 0% (*will not remember any of the rooms*) to 100% (*will remember all of the rooms*). Afterward, the instructions for the study phase began as in Experiment 1.

### Results

As in Experiment 1, we first report the results on source memory, against which metamemory can be compared. Then, we report the results on JOSs, replicating Experiment 1. Finally, we report analyses of the belief judgments to test for an inferential deficit and a utilization deficit of belief.

#### Source memory

We analyzed the responses in the source-monitoring test (Table 1) using the two-high threshold MPT model of source monitoring (Fig. 1; Bayen et al., 1996) in the same way as in Experiment 1. Parameter convergence was good, as was model fit (*T*_1_: *p* = 0.585, *T*_2_: *p* = 0.460). Group did not affect any of the parameters as indicated by all 95% BCIs of the group effects containing zero (Effect_*D*_ = 0.02 [−0.06, 0.10], Effect_*dE*_ = 0.19 [−0.17, 0.57], Effect_*dU*_ = 0.05 [−0.09, 0.20], Effect_*g*_ = 0.04 [−0.09, 0.17], Effect_*b*_ = 0.08 [−0.07, 0.22]). In particular, item memory was affected by neither group nor expectancy. Table 2 shows the full set of parameter estimates. As shown in Fig. 2, both groups showed numerical inconsistency effects. The overall inconsistency effect was reliable, Δ*d* = 0.34 [0.13, 0.56].

#### Metamemory judgments

**JOSs.** We replicated the analyses of JOSs from Experiment 1. Figure 3 shows means and 95% confidence intervals for JOSs. We calculated a 2 × 2 mixed ANOVA with the factors expectancy and belief manipulation on JOSs. As in Experiment 1, there was no main effect of belief manipulation, *F*(1, 119) = 1.10, *p* = 0.296, η_p_^2^ = 0.01, but a main expectancy effect, *F*(1, 119) = 74.91, *p* < 0.001, η_p_^2^ = 0.39, and, critically, a hybrid two-way interaction, *F*(1, 119) = 8.01, *p* = 0.005, η_p_^2^ = 0.06. This interaction indicated that the expectancy effect was stronger in the control group than in the experimental group, even though it was significant in both the control group, *t*(59) = 9.42, *p* < 0.001, *d*_z_ = 1.22, and the experimental group, *t*(60) = 3.69, *p* < 0.001, *d*_z_ = 0.47. Thus, both groups showed illusory expectancy effects on JOSs that were doubly dissociated from the inconsistency effect on source memory. Replicating Experiment 1, the belief manipulation attenuated the expectancy illusion, but not to full remedy.

Also as in Experiment 1, fewer participants fell prone to the illusion in the experimental group than the control group. In the control group, 56 out of 60 participants predicted an expectancy effect, two predicted an inconsistency effect, and another two a null effect. By contrast, in the experimental group, 39 out of 61 participants predicted an expectancy effect, 21 predicted an inconsistency effect, and one a null effect. The proportion of participants who predicted an expectancy effect was smaller in the experimental group than in the control group, *z* = 3.94, *p* < 0.001. Thus, the results for JOSs were very similar to those of Experiment 1.

##### Inferential deficit: Effects of expectancy on belief judgments

The partial persistence of the expectancy illusion in JOSs may be explained by an inferential deficit, a utilization deficit, or a combination of both. We tested for an inferential deficit of belief, that is, whether or not participants in the experimental group adopted the inconsistency belief. Figure 4 shows means and 95% CIs for belief judgments. To analyze belief in the two groups, we calculated a 2 × 2 × 2 mixed ANOVA with the within-subjects factors expectancy and judgment timing (before study, after test) and the between-subjects factor belief manipulation on belief judgments. There was a main effect of judgment timing, *F*(1, 119) = 32.15, *p* < 0.001, η_p_^2^ = 0.21, a two-way interaction between expectancy and group, *F*(1, 119) = 19.76, *p* < 0.001, η_p_^2^ = 0.14, and a two-way interaction between timing and group, *F*(1, 119) = 15.34, *p* < 0.001, η_p_^2^ = 0.11, all of which were qualified by a three-way interaction, *F*(1, 119) = 5.99, *p* = 0.016, η_p_^2^ = 0.05. The other effects were not significant, largest *F* = 1.11, smallest *p* = 0.295. To follow up on the three-way interaction, we conducted separate analyses for the two groups.

In the control group, there was no effect of timing, *F*(1, 59) = 1.54, *p* = 0.219, η_p_^2^ = 0.03. There was a main effect of expectancy, *F*(1, 59) = 13,54, *p* < 0.001, η_p_^2^ = 0.19, which was qualified by a significant two-way interaction, *F*(1, 59) = 4.65, *p* = 0.035, η_p_^2^ = 0.07. Follow-up *t* tests showed that, as expected, the control group believed in an expectancy effect before study (replicating Schaper et al., 2019b), *t*(59) = 4.61, *p* < 0.001, *d*_z_ = 0.60, but not after test (replicating Experiment 1), *t*(59) = 1.07, *p* = 0.290, *d*_z_ = 0.14. Thus, control participants’ initial expectancy belief changed over the course of the experiment, likely due to test experience.

Conversely, in the experimental group, there was a main effect of timing, *F*(1, 60) = 45.77, *p* < 0.001, η_p_^2^ = 0.43, indicating that belief judgments after test were lower than before study. Critically, as expected, there was an inconsistency effect on belief judgments, *F*(1, 60) = 7.45, *p* = 0.008, η_p_^2^ = 0.11, and no interaction,* F*(1, 60) = 1.67, *p* = 0.201, η_p_^2^ = 0.03. This indicates that after receiving the belief manipulation, the experimental group correctly believed in an inconsistency effect and that this belief did not change throughout the experiment.

Despite the experimental group showing an overall inconsistency belief before study, it is nonetheless possible that some participants showed an inferential deficit. To test this, we analyzed the frequencies of participants who held an expectancy belief (i.e., higher belief judgment for expected than unexpected source–item pairs), an inconsistency belief (i.e., higher belief judgment for unexpected than expected source–item pairs), or a null belief (i.e., equal belief judgment for expected and unexpected source–item pairs) before study. In the control group, the majority of participants, 39 out of 60, held an expectancy belief, 10 an inconsistency belief, and 11 a null belief, indicating that even participants’ naïve belief is somewhat heterogeneous (an observation also made by Schaper et al., 2019b). Conversely, in the experimental group, the majority of participants, 34 out of 61, held an inconsistency belief, 13 an expectancy belief, and 14 a null belief. Whereas the proportion of participants who showed a null belief did not differ between groups, *z* = 0.63, *p* = 5.31, there were more participants who showed an expectancy belief in the control group than in the experimental group, *z* = 4.85, *p* < 0.001, and more participants who showed an inconsistency belief in the experimental group than in the control group, *z* = 4.47, *p* < 0.001. Thus, despite the overall effectiveness of the manipulation, 44% of participants in the experimental group failed to adopt the induced inconsistency belief, and, thus, showed an inferential deficit.

##### Utilization deficit: Belief before study moderates the effect of expectancy on JOSs

A utilization deficit regarding belief would be indicated by participants who adopted the inconsistency belief, but did not use it to inform their JOSs. We, therefore, tested whether participants’ belief before study moderated the effect of expectancy on JOSs with a linear mixed regression model (cf. Kenny et al., 2003; Krull & Mackinnon, 2001) using the R packages *lme4* and *lmerTest* (Bates et al., 2015; Kuznetsova et al., 2014) with participants as random effect. Such a moderation is indicated by an interaction between belief before study and expectancy on JOSs (cf. Baron & Kenny, 1986). In this analysis, we wanted to test for a utilization deficit, but not for the already established inferential deficit. We therefore entered only those 34 participants of the experimental group who showed the correct inconsistency belief prior to study and contrasted them with those 39 participants in the control group who showed the incorrect expectancy belief. We refer to these participants as showing group-consistent beliefs. Figure 3 shows the mean JOSs and 95% confidence intervals of these participants who showed group-consistent beliefs. We fitted a model with expectancy (first dummy coded as expected = 1, unexpected = 2, then centered to the participants’ means), group (first dummy coded as 1 = control, 2 = experimental, then centered to the grand mean), belief before study (centered to the grand mean), and all interactions as predictors of JOSs. As a measure of belief before study, we calculated for each participant the difference in the belief judgments for expected and unexpected trials such that positive values indicate an expectancy belief and negative values indicate an inconsistency belief. Unstandardized regression weights and inference statistics are in Table 3 (top half).

**Table 3:** Linear mixed regression model of the effects of group, expectancy, belief, and their interactions on Judgments of Source (JOSs) in Experiment 2

Predictor		Estimate	*SE*	*df*	*t*	*p*
Intercept		59.60	3.12	69	19.13	<.001
Group		8.91	6.17	69	1.45	.153
Expectancy		–19.04	1.09	4595	17.43	<.001
Belief		0.17	0.16	69	1.05	.299
Group × Expectancy		–1.08	2.16	4595	0.50	.619
Group × Belief		0.34	0.31	69	1.10	.275
Expectancy × Belief		–0.46	0.06	4595	8.40	<.001
Group × Expectancy × Belief		–0.64	0.11	4595	5.88	<.001
Group						
Control	Intercept	55.56	2.39	37	23.27	<.001
	Expectancy	–21.25	0.76	2455	28.06	<.001
	Belief	0.01	0.27	37	0.03	.979
	Expectancy × Belief	–0.17	0.08	2455	2.00	.045
Experimental	Intercept	57.97	2.22	32	26.06	<.001
	Expectancy	–4.79	0.88	2140	5.44	<.001
	Belief	0.35	0.17	32	2.09	.045
	Expectancy × Belief	–0.80	0.07	2140	12.22	<.001

Only the data from participants who showed a group-consistent belief entered these analyses. Estimates are unstandardized regression weights. Group: control = 1 (participants did not receive a belief manipulation); experimental = 2 (participants received the belief manipulation before study); expectancy: expected = 1, unexpected = 2; belief: difference between a participant’s belief judgment for expected sources and their belief judgment for unexpected sources before study

As expected, there were an expectancy effect on JOSs (indicated by the significant negative regression weight) and an interaction between expectancy and belief, indicating that belief indeed moderated the effect of expectancy. The results were qualified by a significant three-way interaction, indicating that the relationship between belief and effect of expectancy depended on group. To follow up on this interaction, we calculated one model per group with predictors expectancy and belief (see Table 3, bottom half). In the experimental group, there was a main effect of belief, indicating that the more participants believed in an inconsistency effect, the lower were their JOSs. In both groups, there were significant expectancy effects on JOSs. This indicates that even when belief was taken into account, the expectancy effect remained significant. Furthermore, even those participants who showed an inconsistency belief in the experimental group showed an expectancy effect on JOSs, indicating a utilization deficit. Further critical, there were significant two-way interactions between expectancy and belief in both groups. These interactions indicate that the effect of expectancy depended on belief. In the control group, the more participants believed in an expectancy effect prior to study, the more they showed one in JOSs. In the experimental group, the more participants believed in an inconsistency effect prior to study, the less they showed an expectancy effect in JOSs. The significant three-way interaction indicates that this relationship was stronger in the experimental group, indicating that the experimental group applied their belief more strongly in JOSs than the control group.

Overall, these results show that the partial persistence of the expectancy illusion on JOSs was due to both a partial inferential deficit, in that some participants did not adopt the inconsistency belief, as well as a partial utilization deficit, in that those participants who did show an inconsistency belief still showed the expectancy illusion. Somewhat surprisingly, these participants still used their corrected belief more strongly than the control group used their naïve expectancy belief. That is, participants in the experimental group made an effort to use their corrected belief, but were still prone to the illusion in JOSs. We will discuss potential reasons in the *General discussion*.

## General discussion

In metamemory research, the contributions of experiences and belief to metamemory judgments are of interest. Metamemory illusions and their remedies allow us to test ways to influence and improve metamemory judgments and the bases of judgment formation. However, a change in belief can only remedy a metamemory illusion if the illusion is to a large extent based on belief. In two experiments, we tested the contribution of belief to the metamemory expectancy illusion about source memory by manipulating belief via instruction before study.

### Belief correction partially remedied the metamemory expectancy illusion

Although instruction seems a straightforward way to manipulate belief, its effectiveness at remedying the expectancy illusion was limited in our experiments. We deliberately implemented a strong and elaborate belief manipulation, anticipating that it would strongly affect belief and, consequently, JOSs. This approach was informed by evidence that participants are more likely to accept a belief manipulation if it includes greater detail (Yan et al., 2016). Moreover, a prior experiment showed that a brief belief manipulation merely changed the expectancy belief toward a null belief, but not toward an inconsistency belief (Schaper et al., 2019a, Experiment 4). With the current strong manipulation, we sought to test if a purely belief-based intervention can lead to full remedy of the expectancy illusion. Such a belief-based remedy would be most favorable for future learning, as belief corrections are more likely to generalize to new materials than changes in experiences (Koriat & Bjork, 2006b).

Both experiments showed that despite the strength of the belief manipulation, it led to merely partial, but not full remedy of the illusion. The belief manipulation achieved an attenuation of the expectancy illusion in item-wise JOSs, but did not completely eliminate it. In both experiments, both groups predicted an expectancy effect in their JOSs, even though they actually showed inconsistency effects on source memory, thus both showed the illusion. However, in the experimental groups, fewer participants predicted an expectancy effect on JOSs in the first place and the mean expectancy effect was weaker than in the control group. These findings indicate a partial remedy of the expectancy illusion in JOSs. Thus, our study underscores that misconceptions about (source) memory during study can be strong and difficult to correct (see also Yan et al., 2016).

In Experiment 2, belief judgments before study showed that overall, the belief manipulation was effective at correcting belief. Specifically, as intended, the experimental group showed an overall inconsistency belief, whereas the control group showed an overall expectancy belief. The results suggest, however, that belief was not the only factor driving the expectancy illusion on JOSs. Rather, even when belief was manipulated, fluency experiences resulted in an attenuated, but persistent illusion in JOSs at the moment of study. Thus, belief and experiences contributed jointly to JOSs.

Furthermore, belief judgments after test indicated that test experience additionally affected belief. After test, the control groups showed a null belief which differed from their expectancy belief before study. Even though the control groups did not correctly postdict the inconsistency effect on source memory, experiencing their own source memory during test likely changed metamemory belief to some degree, because they noticed that source memory was not enhanced for expected trials. By contrast, belief did not change with test experience in the experimental group, presumably because this group already believed in an inconsistency effect, and thus, their experiences with their source memory were consistent with this belief.

### Theoretical explanations for the partial persistence of the expectancy illusion

In the following, we discuss in more detail the evidence in support of two possible theoretical reasons for the merely partial success of our attempts to remedy the metamemory expectancy illusion, namely the evidence for an inferential deficit (cf. Hertzog et al., 2009; Mueller et al., 2015) and that for a utilization deficit (cf. Mueller et al., 2015). Our data show that both a partial inferential deficit and a partial utilization deficit contributed jointly to the expectancy illusion.

#### Inferential deficit

The control group of Experiment 2 showed that, at the start of the experiment, participants on average held a naïve erroneous expectancy belief (replicating Schaper et al., 2019b). We sought to change this belief to an accurate inconsistency belief by manipulating belief before study, which was overall successful. Nonetheless, Experiment 2 showed that 44% of participants in the experimental group did not adopt the correct inconsistency belief. Thus, there were individual differences in participants’ inclination to change their belief, with some participants showing an inferential deficit, whereas others adequately changing their belief. Overall, the partial persistence of the expectancy illusion on JOSs is partially explained by an inferential deficit in a substantial subsample of participants.

There are several possible reasons for an inferential deficit that are not mutually exclusive. First, the belief manipulation was externally provided rather than generated by the participants themselves, and participants may have been hesitant to follow such an external manipulation. Second, participants may have maintained an erroneous expectancy or null belief, because, in the current case, the belief manipulation needed not only to induce a new belief (as in Mueller & Dunlosky, 2017), but to fully reverse a pre-existing contrary belief. Participants may have been resistant to fully change this preexisting belief. Third, even if participants fully changed their belief at a general level, they may have been deficient in inferring, from such general belief, a belief about their own individual source memory. Yan et al. (2016) discussed that people assume that they are unique as learners. In their study, a majority of participants identified with the minority of people for whom blocked learning is superior to interleaved learning. Similarly, some of our participants may have inferred that, whereas the majority of people show an inconsistency effect on source memory, they themselves were an exception and would show an expectancy effect. Finally, both the control group and the experimental group showed a similar proportion of participants who showed a null belief, regardless of the belief manipulation. Thus, there seems to be a stable minority of participants who express the belief that expectancy does not affect source memory one way or the other. It is possible that probing for belief judgments may have reactively affected responses (e.g., because participants feel that they are being tricked by the experimenter). Investigating the heterogeneity and individual differences in beliefs about memory is an interesting avenue for future research.

#### Utilization deficit

The partial persistence of the expectancy illusion is further explained by a partial utilization deficit. That is, despite acquiring an accurate metamemory belief, participants used it deficiently. It should be noted in this regard that, in Experiment 2, those participants who held an inconsistency belief in the experimental group utilized their belief more strongly than the control group used their naïve expectancy belief. That is, the belief manipulation not only corrected belief, but also enhanced belief utilization in JOSs. Despite this, the expectancy illusion in JOSs persisted, even in those participants who showed the correct inconsistency belief. This indicates that the corrected belief was underutilized.

A possible reason for the partial utilization deficit is that the belief manipulation had to overcome the experience-based effect of processing fluency in the moment of studying a source–item pair (cf. Koriat, 1997). Expected source–item pairs are more fluently processed than unexpected pairs (as indicated by briefer study times, Schaper et al., 2019b; Sherman et al., 1998) and people equate greater processing fluency with better memory (review by Undorf, 2020). This presumably led to higher JOSs for expected pairs. The current results corroborate strong effects of processing fluency at study, which resulted in an expectancy illusion despite belief manipulation.

The influence of experienced processing fluency on metamemory is thought to be unconscious (e.g., Koriat & Bjork, 2006b; Undorf & Zander, 2017). Therefore, we assume that in our experiments, the direct belief instruction including explicit fluency discounting (i.e., telling participants that expected source–item pairs feel easier to study, but that this contradicts actual source memory) did not affect experiences. Presumably, the experience of processing fluency during study competed with participants’ manipulated belief. Such competing influences may explain why manipulated belief could only attenuate the expectancy effect on item-wise JOSs in most participants. These participants may have failed to fully utilize their accurate belief about source memory in item-wise JOSs, because it contradicted the experiences made at study. About one third of participants, however, were seemingly able to discount their fluency experiences and predicted the inconsistency effect on source memory. Thus, there seem to be individual differences in the utilization deficit as well as the inferential deficit.

Rather than competing experience-based influences, competing different beliefs may also affect metamemory judgments and lead to a deficit in utilizing a correct inconsistency belief. Mueller and Dunlosky (2017) showed that people may hold beliefs about the effects of fluency, which, in turn, affect item-wise JOLs. In our current study, in addition to a naïve expectancy belief, participants may have held the naïve belief that fluently processed information is better remembered than disfluently processed information. Such a belief about fluency coincides with the naïve expectancy belief, but might compete with a corrected belief. We addressed this issue by discounting processing fluency in the belief manipulation. Nonetheless, the expectancy illusion persisted. We, therefore, deem it unlikely that our results are explained by a competing belief about fluency effects. However, future research should discern whether various separate beliefs affect metamemory illusions. In source monitoring, for example, one could separately manipulate the expectancy belief and the fluency belief and observe the according effects on JOSs.

### Conclusion

The experiments presented here show that metamemory illusions and their remedies can provide important information about the theoretical bases of metamemory judgments. Belief plays an important role in the expectancy illusion on JOSs, but experiences in the moment of study also contribute and drive the illusion. Furthermore, there appear to be individual differences in how the belief manipulation affected metamemory, with some participants showing an inferential deficit, others a utilization deficit, and some participants using their corrected belief to make accurate metamemory predictions. Future research should study the relative contributions of belief and experience to metamemory not only at group level, but also at individual level. This may help to discern how metamemory in schema-based source monitoring may be improved more effectively and how such an improvement may be generalized to future study.

The exact contributions of belief and experience to metamemory judgments may vary across memory tasks. However, we presume that the general mechanisms shown in the current study should apply to a variety of metamemory effects. Further research is needed to delineate how these mechanisms operate in different task paradigms and metamemory effects.

## Data Availability

The experiments were not preregistered. Supplementary analyses, materials, data, and the code/syntax used for analyses are provided in the Open Science Framework at https://osf.io/ze3qb.

## Footnotes

1 Note that responses in the source-monitoring test are not only determined by memory, but also by guessing bias (e.g., Kuhlmann et al., 2021). When source memory is measured independently of such bias (via cognitive modeling, see *Results* section), sometimes an inconsistency effect emerges. The emergence of the inconsistency effect partially depends on the strength of the expectancy manipulation: Strong violations of expectations resulted in an inconsistency effect on source memory (e.g., Küppers & Bayen, 2014; Mieth et al., 2021; Schaper & Bayen, 2021; Schaper et al., 2019a, 2019b, 2022, 2023a, 2023b), whereas mild violations of expectations resulted in a null effect on source memory (e.g., Bayen & Kuhlmann, 2011; Bayen et al., 2000; Besken & Gülgöz, 2008; Kuhlmann et al., 2012; Schaper et al., 2023b). The inconsistency effect is best explained by the attention-elaboration account (cf. Brewer & Treyens, 1981; Erdfelder & Bredenkamp, 1998; Friedman, 1979; Loftus & Mackworth, 1978). Presumably, unexpected source–item pairs attract more attention, are elaborated more, and are, therefore, better remembered than expected source–item pairs.

2 Experiment 1 originally included a third group with 60 participants who received only a simple belief manipulation. However, 17 of these participants did not accept this manipulation as indicated by manipulation checks (described in the *Methods* section). The simple belief manipulation was thus inadequate to influence belief and we, therefore, do not report this group here. For interested readers, the full methods and results for this group are reported in detail in the Online Supplementary Material in the Open Science Framework at: https://osf.io/ze3qb/

3 Exclusion of this participant did not change the conclusions.

4 We additionally tested for the interaction between group and expectancy on source memory by calculating one model per group and comparing the inconsistency effect between the groups. Group did not affect the strength of the inconsistency effect, indicated by non-significant interactions in Experiment 1, Δ =.01 [-.45,.43], and Experiment 2, Δ =.15 [-.28,.59].

5 The partial remedy could also result from participants overall predicting a weaker effect of expectancy. A 2 × 2 mixed ANOVA with factors expectancy and belief manipulation among those participants who predicted an expectancy effect in JOSs showed a two-way interaction, *F*(1, 93) = 7.63, *p* =.007, η_p_^2^ =.08, indicating that the expectancy effect was weaker in the experimental group even among those participants who predicted an expectancy effect in JOSs. However, this interaction effect did not replicate in Experiment 2, *F*(1, 93) = 0.02, *p* =.902, η_p_^2^ <.01, and we, therefore, refrain from interpretation.

6 As in Experiment 1, participants in the experimental group completed two manipulation checks after the belief manipulation. One participant initially answered the first question (actual effect on memory) incorrectly, and two different participants initially answered the second question (subjective ease) incorrectly. All three participants responded correctly to both questions after repetition of the manipulation. Exclusion of these participants did not change the overall pattern of results.
